# Failure of A Novel, Rapid Antigen and Antibody Combination Test to Detect Antigen-Positive HIV Infection in African Adults with Early HIV Infection

**DOI:** 10.1371/journal.pone.0037154

**Published:** 2012-06-08

**Authors:** William Kilembe, Michelle Keeling, Etienne Karita, Shabir Lakhi, Paramesh Chetty, Matt A. Price, Heeran Makkan, Mary Latka, Morongwe Likoti, Kenneth Ilukui, Mackenzie Hurlston, Susan Allen, Gwynn Stevens, Eric Hunter

**Affiliations:** 1 Rwanda Zambia HIV Research Group, Kigali, Rwanda, and Lusaka, Kitwe and Ndola, Zambia; 2 Emory University, Atlanta, Georgia, United States of America; 3 International AIDS Vaccine Initiative, New York, New York, United States of America; 4 Aurum Institute for Health Research, Johannesburg, Republic of South Africa; Blood Systems Research Institute, United States of America

## Abstract

**Background:**

Acute HIV infection (prior to antibody seroconversion) represents a high-risk window for HIV transmission. Development of a test to detect acute infection at the point-of-care is urgent.

**Methods:**

Volunteers enrolled in a prospective study of HIV incidence in four African cities, Kigali in Rwanda and Ndola, Kitwe and Lusaka in Zambia, were tested regularly for HIV by rapid antibody test and p24 antigen ELISA. Five subgroups of samples were also tested by the Determine Ag/Ab Combo test 1) Antigen positive, antibody negative (acute infection); 2) Antigen positive, antibody positive; 3) Antigen negative, antibody positive; 4) Antigen negative, antibody negative; and 5) Antigen false positive, antibody negative (HIV uninfected). A sixth group included serial dilutions from a p24 antigen-positive control sample. Combo test results were reported as antigen positive, antibody positive, or both.

**Results:**

Of 34 group 1 samples with VL between 5x105 and >1.5x107 copies/mL (median 3.5x106), 1 (2.9%) was detected by the Combo antigen component, 7 (20.6%) others were positive by the Combo antibody component. No group 2 samples were antigen positive by the Combo test (0/18). Sensitivity of the Combo antigen test was therefore 1.9% (1/52, 95% CI 0.0, 9.9). One false positive Combo antibody result (1/30, 3.3%) was observed in group 4. No false-positive Combo antigen results were observed. The Combo antigen test was positive in group 6 at concentrations of 80 pg/mL, faintly positive at 40 and 20 pg/mL, and negative thereafter. The p24 ELISA antigen test remained positive at 5 pg/mL.

**Conclusions:**

Although the antibody component of the Combo test detected antibodies to HIV earlier than the comparison antibody tests used, less than 2% of the cases of antigen-positive HIV infection were detected by the Combo antigen component. The development of a rapid point-of-care test to diagnose acute HIV infection remains an urgent goal.

## Introduction

Acute HIV infection is the period following transmission of the virus and before antibody seroconversion, during which the risk of HIV transmission is high relative to chronic HIV infection [1]. Diagnosis of acute HIV infection remains a challenge. Acute retroviral syndrome symptoms may not be present, and standard detection methods such as antibody tests will fail to detect the new infection [2], [3]. Diagnostic aides such as risk score algorithms and decision trees can provide some assistance to the clinician who suspects acute HIV infection [4], [5], but will likely miss some cases. The use of HIV RNA tests is recommended in high incidence areas [6], however, the cost, logistics and time needed for these tests makes their widespread use impractical. The development of a point-of-care, rapid test to detect acute HIV infection is therefore important for rapid diagnosis and to allow the timely provision of risk reduction counseling to prevent onward HIV transmission, as well as the provision of treatment where the test and treat strategy becomes available. In addition, in cross sectional surveys of HIV prevalence with adequate sample size, identifying acute cases may allow investigators to estimate HIV incidence [7], [8].

The Determine HIV - 1/2 Ag/Ab Combo test (referred to as the Combo test) is a rapid, visually read, qualitative immunochromatographic test with separate indicators for both antibody and p24 antigen results, allowing to distinguish acute from non-acute HIV infection. We present results generated by testing specimen panels from Rwandan and Zambian adults with or at risk for acquiring HIV infection, and a panel of serially diluted p24 antigen-positive controls.

## Methods

Volunteers are followed in a study of the sexual transmission of HIV in Ndola, Kitwe and Lusaka, Zambia; and Kigali, Rwanda, as described elsewhere [9], [10]; briefly, HIV-uninfected volunteers who are sexually active with a known HIV-infected partner are followed either quarterly or monthly and receive risk reduction counseling and HIV testing at each visit. Routine HIV screening for HIV-uninfected volunteers is done with the Determine rapid test (Abbot Laboratories, Chiba, Japan) and p24 ELISA (Coulter p24 HIV-1 Antigen Assay from January 2002-April 2007 (Beckman Coulter, Inc. Fullerton, California, USA) and the Vironostika HIV-1 p24 Antigen (BioMerieux bv, The Netherlands) from April 2007 to present. Confirmation of a positive Determine result was done with both second and third line rapid tests (Capillus and UniGold, Trinity Biotechnology). Additionally, acute infections were confirmed using polymerase chain reaction (PCR) on the HIV gp41 envelope region followed by genetic sequencing of the virus. Previous work with the p24 ELISA in Africa showed that the recommended assay cut-off led to a high rate of false positive results [11]. We empirically established a more conservative cut-off than either p24 ELISA or Vironostika p24 antigen assay package inserts based on the incidence of false positive results within our cohorts at the time of testing. The rate of false positive fell from 92% to 27% when the cut-offs we chose were used compared to the manufacturers cut-offs (non-published data). Therefore, only samples with results twice that of the recommended assay cut-off value were read as p24 positive with the Vironostika p24 assay, and three times the recommended cut-off for the Coulter p24 assay. Plasma samples were stored at -80?C and did not exceed three freeze thaw cycles as recommended by the Determine HIV ½ Ag/Ab Combo Test package insert. Volunteers with confirmed HIV infection (Ag+/Ab−, Ag+/Ab+, or Ag−/Ab+) were invited to join a study of early HIV infection; subjects were enrolled within 90 days of estimated date of infection (EDI). All volunteers provide informed consent, and these studies were approved by the National Ethics Committee of Rwanda, the University of Zambia Biomedical Research Ethics Committee, and the Emory University Institutional Review Board. All EC/IRBs are registered with the U.S. Office of Human Research Protection.

The testing of specimens was conducted both at the field laboratory in Lusaka, Zambia as well as the laboratory of Professor Eric Hunter at Emory University’s Vaccine Center. The field laboratory is Good Clinical Laboratory Practice (GCLP) accredited by Qualogy Inc., externally audited three times per annum by study sponsors, and participates in quarterly external quality assurance (EQA) testing schemes for all diagnostic testing. The testing of all specimens was done by three trained laboratory scientists who were all in supervisory positions within the organizations and each had greater than 4 years reading and interpreting HIV rapid testing among the Rwandan and Zambian populations. [12].

Plasma samples were retrospectively identified and tested by the Determine HIV-1/2 Ag/Ab Combo test according to the package insert (Combo test, Inverness Medical, Chiba, Japan). All results were read visually by a trained laboratory technician. Samples that produced a line in the results window that appeared to the eye fainter than the control bar were recorded by the technician as “weakly positive” but were considered positive for all analyses, as per the assay package insert. Examples of weakly positive results can be seen in the Inverness Medical report on the Combo test [13] and in [14]. Six groups of samples were tested:

1. Antigen positive, antibody negative samples from acutely HIV-infected volunteers identified between 2002 and 2009, n = 34.
2. Antigen positive, antibody positive samples from volunteers with recent HIV infection. This group includes volunteers with discrepant results across more than one antibody test (i.e., at least one antibody test was positive and at least one was negative), n = 18.
3. Antigen negative, antibody positive samples from volunteers with chronic HIV infection. Includes a convenience sample of volunteers with known HIV infection arriving for routine study visits, n = 28.
4. Antigen negative, antibody negative samples from HIV-uninfected volunteers. This group includes a convenience sample of volunteers who did not show laboratory evidence of HIV infection at this or their subsequent study visit, n = 30.
5. P24 antigen ELISA positive, antibody negative samples from HIV-uninfected volunteers (false positives), n = 25.
6. A serially diluted p24-antigen control sample at a starting concentration of 160 pg/mL (i.e., concentrations of 80, 40, 20, 10, 5, 2.5, 1.25 and 0.0625 pg/mL were tested), n = 1.

All volunteers with positive p24 ELISA results were confirmed as HIV-infected or as false positive by antibody testing at subsequent weekly visits. Confirmatory antibody testing was done using three rapid tests in accordance with both Rwanda and Zambia’s national HIV testing algorithm. Two of three antibody positive tests must be positive to yield a positive HIV antibody test result. A total of 30/34 (88.2%) antigen positive, antibody negative specimens were tested for the presence of HIV RNA (Roche Amplicor, Roche Molecular Systems) on aliquots prepared at the time of Determine HIV-1/2 Ag/Ab Combo testing. To conserve specimens, the antigen positive, antibody negative samples were diluted 20-fold with pooled and confirmed negative p24 antigen and antibody plasma. Therefore, the lower limit of detection of the assay was 8,000 copies/mL. The upper limit of the assay was 15x10^6^ copies/mL. HIV-1 subtyping was done from amplified *gag, pol* or *gp41* sequences using the Recombination Identification Program (http://www.hiv.lanl.gov/content/sequence/RIP/RIP.html) as described previously [15].

The results of the Combo antigen test are compared to the results of the p24 antigen ELISA to calculate test sensitivity. Poisson exact methods were used to calculate 95% confidence intervals. Analyses were conducted with Stata Statistical Software (Release 11, College Station, TX, USA: StataCorp LP) and SAS Statistical Software (*SAS* Institute Inc. 2009, Cary, NC).

## Results

### Study Population

A total of 135 plasma samples from 123 volunteers were tested, including 99 volunteers from Lusaka, Zambia, 14 from the Zambia’s Copperbelt (Kitwe and Ndola), and 10 from Kigali, Rwanda (Table 1). Of the volunteers tested, 78 (63%) were HIV-infected (groups 1-3), and 45 (37%) were HIV-uninfected controls (groups 4 and 5). The volunteers included 58 (47%) women. Nine volunteers provided multiple samples drawn sequentially, including two with HIV infection. One volunteer with HIV infection provided two samples (one Ag+/Ab−; and one Ag+/Ab+) and the second seroconverting volunteer provided two samples (one Ag+/Ab−; and one Ag−/Ab+). The remaining seven volunteers who provided multiple samples were all from group 5 (p24 ELISA false positive volunteers), including four who provided two samples, and three who provided three samples (Table 1). Subtype data were available for 54/78 (69.2%) of the volunteers with HIV infection (Table 1). In Zambia of the 45 for whom subtype was available, 44 (97.8%) were infected with subtype C, and 1 (2.2%) with a subtype A/C recombinant virus. In Rwanda, 7/9 (77.8%) were infected with subtype A1, and 2 (22.2%) with subtype C.

**Table 1:** Study Population Characteristics.

	Group 1:Ag+ Ab−		Group 2:Ag+Ab+		Group 3:Ag−Ab+		Group 4:Ag−Ab−		Group 5:Ag False+Ab−		
Characteristic	N	%	N	%	N	%	N	%	N	%	
Total number of volunteers	34	100	18	100	28	100	30	100	15	100	
**Sex**											
Female	21	61.8	4	23.3	19	67.9	8	26.7	6	40	
Male	13	38.2	14	77.7	9	32.1	22	73.3	9	60	
**Enrolment site**											
Kigali, Rwanda	5	14.7	4	22.2	0	–	0	–	1	7	
Lusaka, Zambia	23	67.7	8	44.4	28	100	30	100	12	80	
Copperbelt, Zambia	6	17.6	6	33.3	0	–	0	–	2	13	
**HIV-1 subtype**											
A1	4	11.8	3	16.7	0	–	0	–	0	–	
C	29	85.3	15	83.3	4	14.3	0	–	0	–	
A/C recombinant	1	2.9	0	–			0	–	0	–	
Missing	0	–	0	–	24	85.7	0	–	0	–	
Not Applicable	0	–	0	–	0	–	30	100	15	100	
**HIV-1 viral load** *											
Number tested	30	88.2	7	38.9	0	–	0	–	0	–	
Median, (IQR)	6.8	(6.2,7.2)	6.2	(6.1,6.8)							
**Number of samples contributed** **											
1	34	100	18	100	28	100	30	100	8	47	
2	0	–	0	–	0	–	0	–	4	33	
3	0	–	0	–	0	–	0	–	3	20	

**Group 1:** Antigen positive, antibody negative acute HIV infection (Ag+Ab−).

**Group 2**: Antigen positive, antibody positive early HIV infection (Ag+Ab+).

**Group 3**: Antigen negative, antibody positive chronic HIV infection (Ag−Ab+).

**Group 4**: Antigen negative, antibody negative volunteers without HIV infection (Ag−Ab−).

**Group 5**: Antigen false positive, antibody negative volunteers without HIV infection (Ag−Ab−).

Ag: p24 antigen, Ab: HIV antibody, weak pos: the respective assay indicator was fainter than the control indicator. This is considered “positive” in our analyses, as per the package insert.

* Log10 transformed viral copies/mL.

** 7/123 volunteers contributed more than one sample to their respective group from different dates (group 5) and 2 volunteers from group 1 contributed a second sample; one to group 2 and the other to group 3.

### Combo Antigen and Antibody Results

Of 34 samples from acutely infected volunteers in group 1 (antigen positive, antibody negative), one sample (2.9%, from a subtype-C infected volunteer) was antigen positive, antibody negative by the Combo test, and seven (20.6%) additional samples were antibody positive but antigen negative (Table 2). Of 18 samples with both detectable p24 antigen and antibody from group 2, no samples were antigen positive and all were antibody positive by the Combo test. Therefore, among all 52 HIV positive samples with a positive antigen ELISA result, the sensitivity of the Combo antigen test was 1.9% (95% confidence interval: 0.0, 9.9). The single antigen positive result from the Combo test was weakly positive, with the antigen band appearing fainter than the control band upon visual inspection.

The univariate summary statistics for p24 antigen concentration among all ELISA identified p24 positives and viral load values are reported in Table 3. The difference in p24 antigen concentration between groups 1 and 2 (Ag+/Ab− and Ag+/Ab+) was not statistically significant p = .1661. Likewise, the concentration of p24 antigen in the 8 specimens that the rapid antigen/antibody was able to identify (one Ag+ and seven Ab+) versus the 26 ELISA p24 positive specimens that tested negative on the rapid assay did not differ with significance, p = .9031.

**Table 2:** Performance of the Determine Ag/Ab Combo test.

	Group 1:Ag+ Ab−		Group 2:Ag+Ab+		Group 3:Ag−Ab+		Group 4:Ag−Ab−		Group 5: Ag False+ Ab−	
Determine Ag/AbCombo test results	N	%	N	%	N	%	N	%	N	%
Total number of samples	34	100	18	100	28	100	30	100	25	100
Ag negative, Ab negative	26	76.5	0	0	0	0	29	96.7	25	100
Ag positive, Ab positive	0	0	0	0	0	0	0	0	0	0
Ag positive, Ab negative	0	0	0	0	0	0	0	0	0	0
Ag weak pos, Ab negative	1	2.9	0	0	0	0	0	0	0	0
Ag negative, Ab positive	1	2.9	15	83.3	28	100	0	0	0	0
Ag negative, Ab weak pos	6	17.6	3	16.7	0	0	1	3.3	0	0

Group 1: Antigen positive, antibody negative acute HIV infection (Ag+Ab−).

Group 2: Antigen positive, antibody positive early HIV infection (Ag+Ab+).

Group 3: Antigen negative, antibody positive chronic HIV infection (Ag−Ab+).

Group 4: Antigen negative, antibody negative volunteers without HIV infection (Ag−Ab−).

Group 5: Antigen false positive, antibody negative volunteers without HIV infection (Ag−Ab−).

Ag: p24 antigen, Ab: HIV antibody, weak pos: the respective assay indicator was fainter than the control indicator. This is considered “positive” in our analyses, as per the package insert.

**Table 3:** ELISA p24 Antigen Concentration and Viral Load Summary Statistics.

	p24 Ag Concentration (pg/ml)					
Summary Statistics	Groups 1 & 2	Group 1	Group 1 SubsetRapid Test NEG	Group 1 Subset RapidTest POS (Ag or Ab)	Group 2	Viral Load (copies/ml)
**N**	52	34	26	8	18	37
**Mean**	126.01	138.7	136.2	146.8	102.1	7,013,211
**Median**	94.1	140.4	140.4	162	50.4	6,316,740
**IQR (75%,25%)**	(201.2,32.7)	(203.5,48.4)	(193,51)	(267.8,14.4)	(152.7,15.7)	(12950000, 1706540)
**Min,Max**	(8.2,457.8)	(8.2,457.9)	(14.2,457.9)	(8.2,277.7)	(10.4,351.2)	(545720,15000001*)

* Upper limit of detection for the VL assay  = 15,000,000, any observation that exceeded 15,000,000 recorded as 15,000,001.

Group 1: Antigen positive, antibody negative i.e. acute HIV infection (Ag+ Ab−).

Group 2: Antigen positive, antibody positive i.e. early HIV infection (Ag+ Ab+).

Viral load data were available from 37 (71.2%) of the group 1 and 2 samples. The median HIV RNA concentration was 3.5x10^6^ copies/mL, with 8 values above the assay upper limit of 15x10^6^ copies/mL. The sample positive by Combo antigen had a viral load of 1.1x10^7^ copies/mL and was higher than 66% of the group 1 and 2 samples with viral load data. There was a strong correlation between p24 antigen concentration and viral load for Ag+Ab− individuals (0.741, p<.0001) as well as combined Ag+Ab− and Ag+Ab+ (0.722, p<.0001).

None of the group 4 (n = 30) or group 5 (n = 25) samples from volunteers without HIV infection were antigen-positive by the Combo test. However one group 4 sample (3.3%) was antibody-positive by the Combo test, a false positive result. In a comparison of the Combo test antigen component to the ELISA antigen test, both assays were run against serially diluted p24 antigen control provided in the Vironostika HIV-1 Antigen ELISA kit; in which the positive control is serial diluted with the kit’s negative control per testing instructions (group 6). The reported optical density of the serial p24 dilution was the average of duplicate runs. The Combo antigen test remained positive to p24 antigen concentrations of 20 pg/mL and the ELISA remained positive to 5pg/mL (Table 4).

**Table 4:** Comparison of the p24 ELISA (Vironostika) and Combo test (antigen component) against serially diluted p24 antigen-positive controls.

Positive controldilution(pg/mL)	p24 ELISA OD*	p24 ELISAResult	Combo rapidtest result at60 minutes
80	1.454	Positive	Positive
40	0.737	Positive	Positive
20	0.4	Positive	Positive
10	0.226	Positive	Negative
5	0.137	Positive	Negative
2.5	0.083	Negative	Negative
1.25	0.053	Negative	Negative
0.0625	0.043	Negative	Negative

* Average OD (Optical Density) of two runs. An OD ≥0.10 is considered a positive result.

## Discussion

In this prospective cohort of African adults infected with or at high risk for HIV infection, the Determine Ag/Ab Combo test detected only 8 of 34 (23.5%) persons with acute HIV infection. Only one of these 8 positive Combo test results was scored as antigen-positive, the remaining 7 were antibody-positive. Also, the antigen component of the Combo test did not detect any of the 18 samples that were antigen- and antibody-positive in volunteers with early HIV infection, and when tested against serially diluted p24 antigen-positive controls, was not as sensitive as the p24 ELISA. While the antibody component of the Combo test was able to detect antibodies to HIV before the rapid HIV tests used in this report, the antigen component of the Combo test was much less sensitive than the p24 antigen ELISA in detecting acute infection in these subtype A and C plasma samples. The Combo test package insert reports two analytical sensitivity studies one which used an HIV Ag panel from the French Blood Establishment and the second used purified HIV-1 p24 native protein. The reported lower limits of p24 antigen concentration that could be detected by the Combo assay were 25 pg/ml and 12.5 pg/ml respectively [16]. Twenty one percent (11/52) of the acute infections we identified with the p24 Antigen ELISA had concentrations less than 25 pg/ml but of these 11, only 2 had p24 Antigen concentrations less than 12 pg/ml and theoretically should have been picked up by the Combo assay. The sole acute infection that was detected by the Combo assay had a p24 antigen concentration of 248.3 pg/ml. Recent work in Malawi, where the HIV epidemic is primarily subtype C, also found that the Combo test failed to detect acute HIV infection. The authors report that 8 cases of acute HIV infection were missed by the antigen component of the Combo test (although 2 were detected by the antibody component), and that 14 false positive Combo antigen results were observed in 838 HIV-uninfected persons, for a sensitivity of 0.00 and a specificity of 0.98 [17]. A report from France to evaluate the sensitivity of five rapid HIV tests in a health care setting found the Combo test did not detect p24 antigen in two volunteers with recent HIV infection. The concentration of p24 antigen in the plasma of one volunteer was 380pg/mL, and undetectable in the other [18]. In contrast, the assay has been tested in San Francisco, and correctly identified all persons with chronic infection, and 7/9 persons with acute HIV infection [19]. Another recent report found the sensitivity of the Combo test in a bank of acute HIV infection specimens from Holland to be 86.6% (32/39 samples, 95% CI: 76.0, 93.7). However two of the 32 samples were reactive in the antibody component only and no subtype or demographic data are given. The authors of this work also report being able to detect antigen in cell supernatants from subtypes A, B, C, D, CRF_01AE, CRF01_AG, G, G/H, J, G/A, and K (3–5 samples of each) diluted in negative human plasma, however the concentration of p24 antigen in each sample was not reported [20].

While the package insert for the Combo test states that specimens that have been freeze-thawed more than three times should not be used for testing, a majority of our samples tested had not been thawed more than once. Moreover, the specimen in this report that was antigen-positive by both p24 antigen ELISA and Combo test was 3.5 years old at the time of testing. None of the 31 more recent group 1 or 2 samples were detected by the antigen component of the Combo test, including four samples that were drawn within 2 months of testing. Finally, all viral load assays on group 1 and 2 samples were performed on aliquots processed in parallel to those tested in the Combo test, and so it is unlikely that freeze-thawing of samples contributed to the lack of reactivity in this assay.

Reduced performance may have been related to the circulating strains of HIV. Although this assay reportedly detected p24 antigen in cell supernatant of viral cultures of non-B subtype HIV [20], we were unable to reproduce these results using plasma samples from study volunteers infected with subtype A and subtype C HIV-1. The single positive antigen results from the Combo test was from a volunteer infected with subtype C HIV-1.

Strengths of this report include the prospective study design, and the availability of HIV PCR results. All volunteers were enrolled in a prospective study of HIV incidence in HIV-discordant couples. At each visit, volunteers are given risk-reduction counseling and testing for HIV infection. Despite counseling, HIV transmission continued to occur albeit at a reduced frequency and monthly follow-up facilitated detection of HIV infection prior to HIV seroconversions [21]. Because all volunteers were followed up, we were able to confirm their HIV status at follow up visits. A number of instances of p24 positive ELISA results were observed on repeated-testing of volunteers without HIV infection (manuscript in preparation). These provided the samples for group 5. All volunteers with acute or early HIV infection were eventually confirmed with detection of HIV by PCR, and 81% of volunteers from group 1 and 2 had viral load data available from that visit. Viral load and p24 antigen concentrations are typically positively correlated [22], [23]. Because group 1 and 2 samples came from acute and early HIV infection, viral load was very high (frequently exceeding 10^7^ copies/mL). Indeed, one third of the samples with data had a higher viral load than the sample that was Combo antigen positive, and yet none of these scored positive.

Although the package insert instructs the user to score a faintly visible band as a positive result, these weak positive results may be difficult to read [13] and training is important. The only antigen positive result we observed was fainter than the control band, as was 10 of 54 (18.5%) of the antibody positive results we reported. In our experience with HIV antibody rapid tests, a weak positive result can be indicative of a false positive test result [12], and 1 of 10 (10%) of the observed weak positive antibody results in this report were false positive; 44 of 44 (100%) of the Combo antibody positives without a weakly positive band were true seroconversions. Another study considers weak positive as a doubtful result and used an algorithm that required retesting of samples. This study found that of the 2.3% of the results classified as weakly positive results, 46% were resolved as negative [12].

We found the Combo antigen component of the Determine test to be unsatisfactory as a point-of-care test for the rapid diagnosis of acute HIV infection in this predominantly subtype C sample of study volunteers. Given the importance of proper diagnosis of acute HIV infection, a critical time of increased infectivity, the development of rapid diagnosis tools remains an urgent priority.
